# Intimate partner violence victimisation and its association with maternal parenting (the 2015 Pelotas [Brazil] Birth Cohort): a prospective cohort study

**DOI:** 10.1016/S2214-109X(23)00282-6

**Published:** 2023-08-15

**Authors:** Carolina V N Coll, Aluisio J D Barros, Alan Stein, Karen Devries, Romina Buffarini, Lynne Murray, Adriane Arteche, Tiago N Munhoz, Mariângela F Silveira, Joseph Murray

**Affiliations:** a Postgraduate Program in Epidemiology, Federal University of Pelotas, Pelotas, Brazil; b Human Development and Violence Research Centre, Federal University of Pelotas, Pelotas, Brazil; c School of Psychology, Federal University of Pelotas, Pelotas, Brazil; d Department of Psychiatry, University of Oxford, Oxford, UK; e London School of Hygiene & Tropical Medicine, London, UK; f Department of Global Health and Development, University of Reading, Reading, UK; g Department of Psychology, Pontifical Catholic University of Rio Grande do Sul, Porto Alegre, Brazil

## Abstract

**Background:**

Intimate partner violence (IPV) is highly prevalent in low-income and middle-income countries and has been a major obstacle towards reaching global health targets for women and children. We aimed to investigate cross-sectional and longitudinal associations between IPV victimisation and maternal parenting practices of young children in a population-based birth cohort study in Brazil.

**Methods:**

The 2015 Pelotas Birth Cohort is an ongoing, prospective cohort, including all hospital births occurring between Jan 1 and Dec 31, 2015, in the city of Pelotas, Brazil. When children were aged 4 years, mothers reported on emotional, physical, and sexual IPV victimisation in the past 12 months. Parenting outcomes were assessed through filming the mother and child in interactive tasks at age 4 years and maternal interviews at ages 4 years and 6–7 years. Interactive tasks were filmed at the Centre for Epidemiological Research facilities. Directly observed outcomes included negative (eg, coercive) and positive (eg, sensitivity and reciprocity) parenting interactions independently coded by a team of psychologists. Self-reported parenting was measured using the subscales on quality of parent–child relationship, positive encouragement, parental consistency, and coercive behaviour of the Parenting and Family Adjustment Scales questionnaire. Unadjusted and adjusted linear regression analyses were performed to assess the associations.

**Findings:**

Of the 4275 livebirths enrolled in the cohort, 3730 mother–child dyads were included in our analytical sample at age 4 years and 3292 at age 6–7 years. After adjusting for all potential confounders, emotional IPV and physical or sexual IPV were associated with the following self-reported parenting outcomes: poor parent–child relationship quality (emotional IPV: p=0·011), lower parental consistency (emotional IPV: p<0·001, physical or sexual IPV: p=0·0053), and more coercive behaviour (emotional IPV: p<0·001, physical or sexual IPV: p=0·0071) at age 4 years. Associations were not observed for self-reported positive encouragement and filmed parenting outcomes in fully adjusted models. Longitudinally, IPV at age 4 years predicted similar outcomes when children were aged 6–7 years.

**Interpretation:**

In this large cohort study, maternal IPV victimisation was consistently associated with poorer parent–child relationship, decreased parental consistency, and increased harsh parenting reported by mothers of young children. As well as initiatives to prevent IPV, parenting interventions focused on supporting the capacity of caregivers to provide nurturing care delivered at key stages early in the life course are crucial.

**Funding:**

Wellcome Trust.

**Translation:**

For the Portuguese translation of the abstract see Supplementary Materials section.

## Introduction

Violence against women by male partners is a global public health concern affecting the lives of millions of women and their children.^1^ Intimate partner violence (IPV) victimisation, which can include harmful behaviours that are physical, sexual, or psychological (or a combination of these behaviour types), undermines maternal wellbeing and negatively affects mother–child interactions and parenting practices that are linked to healthy childhood development (eg, warmth, responsive parenting, sensitive communication, protection, and stimulation).^2^, ^3^, ^4^ For children, exposure to early adversity, including violence in the home, has further implications for lifecourse outcomes such as poor health and quality of life. This finding has been a particular concern in low-income and middle-income countries (LMICs) where children are more likely to be exposed to multiple risk factors for poor early development.^5^

Although there is a considerable evidence base on the harmful effects of parental conflict on parent–child interactions, which is implicated in important theories such as the spillover hypothesis—suggesting an interdependency between the feelings experienced across different family relationships^6^—the specific effect of IPV (as opposed to other forms of conflict) on parenting has been a more recent focus of interest. Two systematic reviews on caregiving in the context of IPV provide substantive evidence of associations with a range of poor parenting practices of mothers experiencing IPV.^4^, ^7^ In 2018, a meta-analysis by Chiesa and colleagues^7^ showed modest effect sizes for the association between IPV victimisation and increased physical aggression and neglect among female caregivers of children aged 11 years or younger. Associations of IPV experience with diminished engagement and connectedness and lack of effective parenting skills (eg, problem solving, consistency, and protectiveness) were also highlighted. However, a high level of heterogeneity across studies and many methodological challenges were observed. All studies were conducted in the USA, few were prospective, and most samples were relatively narrowly focused (eg, recruited from the welfare system or shelters for women victims of violence), limiting generalisability to the general population.^7^ More recently, Souza and colleagues^4^ conducted a scoping review of 64 quantitative, qualitative, and mixed-method studies on mothering in the context of IPV and found that IPV was related to a decrease in nurturing interactions and communication overall, and often led to an increase in aggression towards children.

**Research in context**

**Evidence before this study**

We found two systematic reviews on maternal intimate partner violence (IPV) victimisation and parenting practices. The first review was carried out by Chiesa and colleagues in 2018 and included 33 quantitative studies with modest effect sizes for the association between IPV victimisation and harmful parenting practices (such as physical aggression and neglect), but high levels of heterogeneity across studies. The second review was conducted by Sousa and colleagues in 2021 and integrated findings from 64 studies including quantitative, qualitative, and mixed methods, to understand more deeply how mothers experience and respond to the effects of IPV on their parenting. The analysis suggested that IPV can undermine parenting, often through its effects on mothers’ sense of efficacy and mental health. Both reviews highlighted several pervasive methodological problems of the studies, which used narrowly focused samples without clear temporal ordering of variables; moreover, most studies had been conducted in high-income countries (mainly the USA).

**Added value of this study**

This is the first prospective population-based birth cohort study to investigate the associations between maternal IPV victimisation and parenting outcomes across early to mid childhood in low-income and middle-income countries (LMICs). In addition to examining associations with harsh parenting and physical punishment, which have been the focus of the few studies carried out in LMICs, we evaluated the effects of IPV on a range of positive aspects of maternal parenting relevant to child development. This study adds to the scarce data employing observed measures of parenting that provide both a more independent assessment and an investigation of more subtle aspects of parent–child interactions, in contrast to general behaviours, which are commonly reported in interviews and self-report questionnaires. We were also able to examine the potential distinct effects of emotional IPV and physical or sexual IPV on parenting, which is not commonly seen in the field. We found that IPV victimisation experienced by the mother (either emotional IPV or physical or sexual IPV) was consistently negatively associated with parent–child relationship quality and parental consistency, and was positively associated with coercive behaviour when children were aged 4 years and 6–7 years.

**Implications of all the available evidence**

Our findings contribute strong evidence to the literature on the adverse effects of violence against women, and underscore the important role that IPV prevention strategies can play in enhancing the quality of mother–child interactions. As well as initiatives to prevent IPV, parenting interventions focused on supporting the capacity of caregivers to provide nurturing care (eg, reducing harsh parenting and increasing positive parenting behaviours) delivered at key stages early in the life course are crucial for improving maternal and child wellbeing.

Scarce evidence is available on the links between IPV and broad maternal parenting practices in LMICs. Previous cross-sectional studies have focused on the specific association between IPV and violent discipline of children, with most showing positive associations.^8^, ^9^, ^10^, ^11^ In a multicountry study conducted in the Asia-Pacific region, women's harsh parenting practices were strongly driven by their own experiences of trauma and abuse through their life course, including child maltreatment and physical IPV.^11^ Worldwide, in both high-income countries and LMICs, there is a dearth of studies using observational methods of parenting that provide more independent assessment and measurement of more subtle aspects of parent–child interactions, in contrast to general behaviours that are commonly assessed in interviews and self-report questionnaires.^12^

Theoretical mechanisms linking IPV with compromised parenting include mental health difficulties arising from IPV, diminished maternal confidence and sense of identity in their role as caregivers, and violent partners controlling women's capacities and resources to care for their children.^4^ However, households experiencing multiple disadvantages (eg, poverty, low education, and maternal depressive symptoms) are at risk of both IPV^13^ and compromised parenting,^5^ representing important potential confounds in the association. In the 2015 Pelotas Birth Cohort Study in Brazil, 22% of approximately 4000 mothers reported experiencing IPV in the past 12 months when their children were aged 4 years.^13^ We aimed to investigate the association between IPV victimisation and a broad range of maternal parenting practices in young childhood among participants from this population-based cohort, at ages 4 years and 6–7 years, using both directly observed and self-reported measures of parenting, and to explore the potential distinct effects of exposure to emotional IPV versus physical or sexual IPV. We hypothesised that IPV is associated with decreased positive parenting behaviours (eg, sensitivity and consistency) and increased harsh parenting.

## Methods

### Study setting and participants

The 2015 Pelotas (Brazil) Birth Cohort Study is a population-based, prospective cohort study, including all children born in 2015 in Pelotas, a city in southern Brazil with a population of approximately 340 000 people. Of the 4333 livebirths that took place in the city maternity hospitals from Jan 1 to Dec 31, 2015 (approximately 99% of all births in Pelotas occurred in hospitals), 4275 (99%) children were enrolled in the cohort and have been followed up since then. At the last follow-up, when children were aged 6–7 years, the response rate was 92%. Detailed methods of the cohort study and each follow-up have been published elsewhere.^14^ The study protocol was approved by the School of Physical Education Ethics Committee at the Federal University of Pelotas (CAAE registration number: 26746414.5.0000.5313) and psychosocial assessments done at age 4 years, including assessments of violence, were approved by the Ethics Committee of the Faculty of Medicine, Federal University of Pelotas (CAAE registration number: 03837318.6.0000.5317). Written informed consent was obtained from parents or guardians at each visit. Violence measurements were conducted in confidential interviews and psychological support was available when positive responses were given. In these cases, the psychologists provided brief counselling and gave information about appropriate community support services.

### Procedures

Information on recent IPV victimisation for mothers was collected through confidential interviews conducted by trained female interviewers using the WHO questionnaire at the 4 years follow-up. The instrument was previously validated in Brazil,^15^ and it is built on the tradition of the Conflict Tactics Scale questionnaire in which respondents are asked about their experience of specific acts of physical (six items), sexual (three items), and emotional violence (four items) perpetrated by current or former intimate partners in the 12 months preceding the interview.^15^ Each domain was scored positively if at least one of its constituent items was scored yes, independently of frequency (which was not assessed in our sample). We created two dichotomous variables: emotional IPV and physical or sexual IPV. We modelled IPV and parenting associations using a clean reference group of no physical, sexual, or emotional IPV. This strategy followed the recommendations of Heise and colleagues^16^ to remove all other forms of violence from our exposure measure when studying IPV as an explanatory variable (eg, when estimating the effects of physical or sexual IPV alone, women experiencing emotional IPV were also excluded from the reference group used in the analysis, as leaving women in who have experienced emotional IPV would probably attenuate the effect of physical or sexual IPV on the outcomes since types of violence frequently co-occur). The overlap of types of IPV in our sample can be checked in appendix 2 (p 12).

Potential confounders included maternal and family characteristics, which were assessed from the time of delivery (perinatal visit) until children were aged 4 years. A representation of the assumptions about the relationship between variables is presented in appendix 2 (p 13). Mothers reported on adverse childhood experiences (ACEs), experienced up to age 18 years, using a shortened version of the WHO ACE-IQ questionnaire at the 4 years follow-up.^17^ ACEs included emotional abuse, physical abuse, sexual abuse, violence against household members, living with substance users, living with household members who were mentally ill or suicidal, living with household members who were incarcerated, and physical neglect and parental loss or divorce. A score ranging from 0 to 9 was created. Maternal schooling, age, and parity was assessed at the time of delivery and recorded as complete years of formal education, age in years, and number of previous livebirths. Family income was assessed at 2 years and was recorded as the total amount earned by family members in the month before the interview (collected as a continuous variable in the local currency [Brazilian reais]). Maternal depressive symptoms were measured at 2 years using the Edinburgh Postnatal Depression Scale.^18^, ^19^ Mothers reported about symptoms experienced in the past week (score ranging from 0 to 30). Partner status was assessed at 2 years. Mothers were asked if they currently had a partner. Child's sex was measured at delivery and age in months was measured at each follow-up.

### Outcomes

Maternal parenting practices examined in the current study are described in detail in appendix 2 (pp 7–10). Assessments were conducted by trained interviewers at the university research centre. Coding of observational measures was completed by a central independent team of psychologists not involved in the data collection, with approximately 400 assessments (10%) double-coded as part of a quality control process and to assess inter-rater reliability. Self-reported parenting behaviours in the past month were assessed using a brief version of the Parenting and Family Adjustment Scales (PAFAS) proposed by Sanders and colleagues^20^ and further supported by a validation study carried out in Brazil.^21^ Questions were read aloud to the mothers given literacy variations in the sample. Positive parenting outcomes included a combined measure of responsive interactions,^22^ sensitivity,^23^, ^24^ reciprocity,^23^, ^24^ and guidance behaviours^17^ of the mother during filmed interactions with the child and, from PAFAS, the parent–child relationship, positive encouragement, and parental consistency subscales.^20^ Harsh parenting was assessed by direct observations of maternal verbal and physical coercion of the child,^17^ and the coercion subscale of the PAFAS questionnaire.^20^

### Statistical analysis

Descriptive statistics are provided in terms of absolute and relative frequencies for categorical variables, and means and SD, medians and IQR, and ranges (minimum and maximum values) for continuous variables. Outcomes were standardised (mean 0, SD 1) to allow comparability between measures of associations for all parenting variables. Multiple linear regression analyses were performed to assess the association between IPV and maternal parenting outcomes. Three models were included for each outcome: unadjusted results (model 1), results adjusted for all confounders except previous depressive symptoms (model 2), and fully adjusted results controlling for all the potential confounders (model 3). The option to have two separate adjusted models (with and without previous depressive symptoms) was due to the potential for depression to be a consequence of persistent IPV exposure, which was not assessed in the present study as we did not have previous measures of IPV (before the 4 years assessment).^25^ CIs were calculated using robust adjustment of variance. Separate models were estimated for emotional and physical or sexual IPV. In the adjusted models we further controlled for inclusion of some cohort families in parenting programmes in the city (ACT: Raising Safe Kids, a book-sharing programme in The Pelotas Trial of Parenting Interventions for Aggression Trial,^26^ and the home visiting programme, Primeira Infância Melhor^27^), which target more vulnerable families and aim to influence parenting practices. We controlled for the inclusion of some cohort families in parenting programmes thinking that these mothers might be more exposed to IPV, although this was not an inclusion criterion for these interventions. We guarded against inflated false positive (type I errors) from multiple testing by performing the analyses on the entire sample, and then applying Benjamini-Hochberg adjustment with a false discovery rate of 10%.^28^ A sensitivity analysis exploring a dose–response relationship between the number of abusive acts of emotional IPV experienced by mothers and parenting outcomes was carried out given that there is still no international consensus on definitions and what level of such acts constitutes abuse.^1^, ^16^

### Role of the funding source

The funders of the study had no role in study design, data collection, data analysis, data interpretation, or writing of the report.

## Results

**Figure 1:**
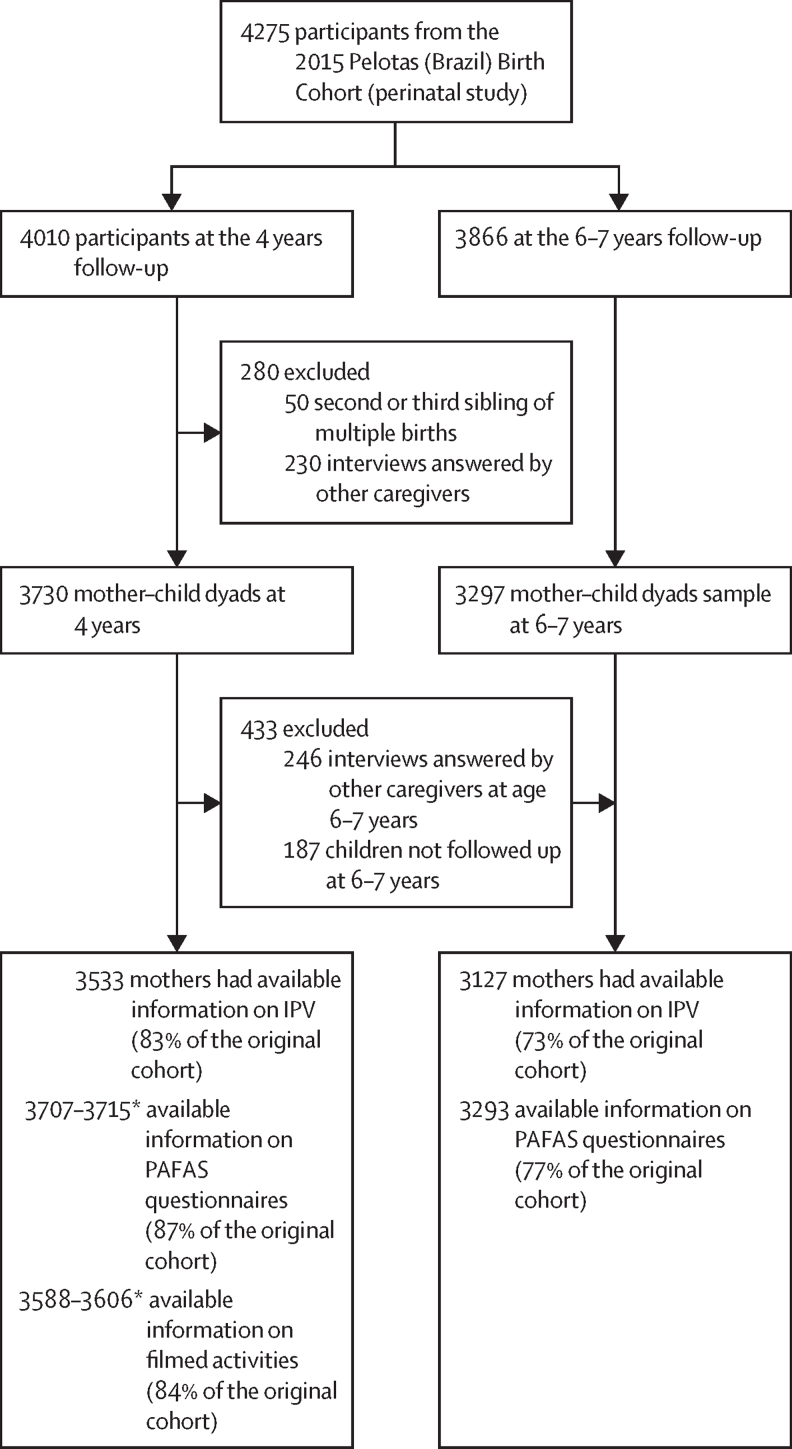
Study participants flowchart and analytic sample assessment at 4 and 6–7 years follow-ups. 2015 Pelotas (Brazil) Birth Cohort IPV=intimate partner violence. PAFAS= Parenting and Family Adjustment Scales. *Complete data vary depending on outcome.

Of the 4275 participants who were enrolled in the perinatal study between Jan 1 and Dec 31, 2015 (99% of all eligible births in the reference year), 3730 mother–child dyads comprised the analytical sample at age 4 years and 3292 at age 6–7 years (figure 1). Mean age of the cohort was 3·8 (SD 0·2) at the 4 years follow-up and 6·8 (SD 0·3) at the 6–7 years follow-up*.* Table 1 shows sample characteristics. More than half of the children were boys (50·6%). About a third of mothers had less than 8 years of formal education, about two-thirds were aged between 20 and 34 years, and half were first-time mothers. Risk of depression assessed when the child was aged 2 years was present for 28·3% of mothers. At the 4 years follow up, 15·6% of the mothers reported 4 or more ACEs. Nearly a quarter of mothers (21·9%) reported emotional IPV, whereas 9·4% reported physical or sexual IPV. Descriptive statistics of maternal parenting outcomes in the total sample and according to IPV exposure status are presented in appendix 2 (pp 10–11).

**Table 1:** Characteristics of the sample and comparison according to IPV victimisation status at age 4 years of the children (previous 12 months), 2015 Pelotas Birth Cohort Study

	**Overall (n=3730)**	**No IPV (n=2726)**	**Emotional IPV (n=766)**	**Physical or sexual IPV (n=283)**
**Child's sex**				
Boy	1886 (51%)	1381 (51%)	384 (50%)	139 (49%)
Girl	1844 (49%)	1345 (49%)	382 (50%)	144 (51%)
**Family income, quintiles**				
Q1 (poorest)	730/3728 (20%)	460/2725 (17%)	186/765 (24%)	83 (29%)
Q2	773/3728 (21%)	559/2725 (21%)	172/765 (22%)	71 (25%)
Q3	757/3728 (20%)	548/2725 (20%)	162/765 (21%)	60 (21%)
Q4	755/3728 (20%)	580/2725 (21%)	136/765 (18%)	38 (13%)
Q5 (richest)	713/3728 (19%)	578/2725 (21%)	109/765 (14%)	31 (11%)
**Maternal education, years**				
0–4	324/3729 (9%)	217/2725 (8%)	78 (10%)	39 (14%)
5–8	959/3729 (26%)	657/2725 (24%)	224 (29%)	99 (35%)
9–11	1311/3729 (35%)	969/2725 (36%)	259 (34%)	88 (31%)
≥12	1135/3729 (30%)	882/2725 (32%)	205 (27%)	57 (20%)
**Maternal age, years**				
<20	526/3729 (14%)	372/2725 (14%)	122 (16%)	53 (19%)
20–34	2661/3729 (71%)	1957/2725 (72%)	536 (70%)	198 (70%)
≥35	542/3729 (15%)	396/2725 (15%)	108 (14%)	32 (11%)
**Mother's parity**				
1	1865/3728 (50%)	1408/2725 (52%)	350/765 (46%)	111/282 (39%)
2	1160/3728 (31%)	855/2725 (31%)	232/765 (30%)	90/282 (32%)
3 or more	703/3728 (19%)	462/2725 (17%)	183/765 (24%)	81/282 (29%)
**Skin colour of the mother**				
White	2675/3726 (72%)	1995/2723 (73%)	525/765 (69%)	190/282 (67%)
Brown	491/3726 (13%)	340/2723 (12%)	114/765 (15%)	44/282 (16%)
Black	560/3726 (15%)	387/2723 (14%)	123/765 (16%)	48/282 (17%)
**Maternal risk of depression (EPDS ≥10)**				
No	2514/3504 (72%)	1979/2572 (77%)	394/712 (55%)	129/268 (48%)
Yes	990/3504 (28%)	592/2572 (23%)	318/712 (45%)	139/268 (52%)
**ACEs**				
0	911/3712 (25%)	757/2722 (28%)	97 (13%)	31 (11%)
1 to 3	2223/3712 (60%)	1653/2722 (61%)	451 (59%)	151 (53%)
≥4	578/3712 (16%)	312/2722 (11%)	218 (28%)	101 (36%)
**PIM intervention**				
No	3448/3716 (93%)	2529/2720 (93%)	702/762 (92%)	256/281 (91%)
Yes	268/3716 (7%)	191/2720 (7%)	60/762 (8%)	25/281 (9%)
**PIÁ intervention**				
No	3487 (93%)	2568/2726 (94%)	708 (92%)	255 (90%)
Yes	243 (7%)	158/2726 (6%)	58 (8%)	28 (10%)
**Mother's partner is biological father of the child**				
No	325/2985 (11%)	250/2373 (11%)	67/558 (12%)	27/168 (16%)
Yes	2660/2985 (89·1%)	2123/2373 (89%)	491/558 (88%)	141/168 (84%)
**IPV (past 12 months)**				
Emotional	766/3532 (22%)	NA	NA	NA
Physical or sexual*	283/3533 (8%)	NA	NA	NA

All variables have <1·0% missing data, except for risk of maternal depression (6·0%) and IPV (5·3%). ACEs=adverse childhood experiences. EPDS=Edinburgh Postnatal Depression Scale. IPV=intimate partner violence. NA=not applicable. PIÁ=The Pelotas Trial of Parenting Interventions for Aggression (which included ACT: Raising Safe Kids and a book-sharing programme). PIM=Primeira Infancia Melhor (Better First Childhood).

* Physical IPV: 262 (8·8%); sexual IPV: 55 (2·0%).

**Figure 2:**
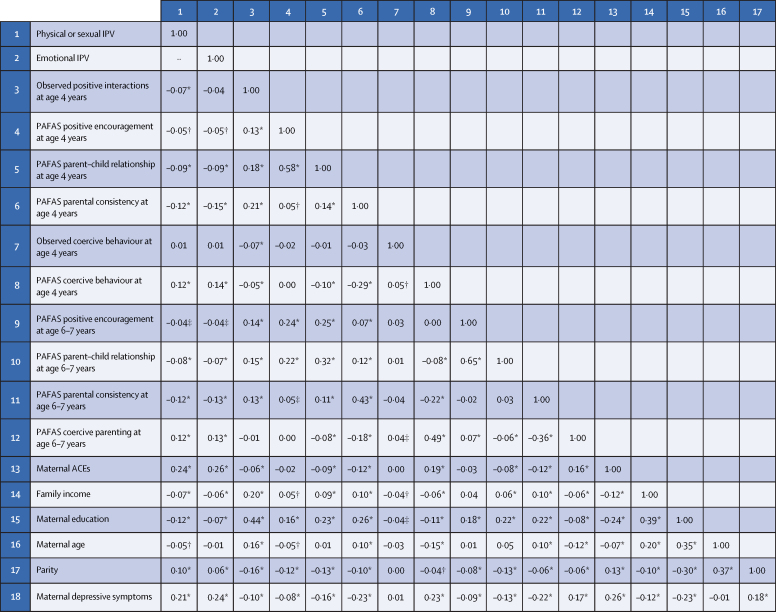
Correlation between exposures, outcomes, and potential confounding variables. 2015 Pelotas (Brazil) Birth Cohort Pearson correlation. Correlation coefficients can range from –1 to +1. ACEs=adverse childhood experiences. IPV=intimate partner violence. PAFAS=Parenting and Family Adjustment Scales. *p<0·001. †p<0·01. ‡p<0·05.

Correlations between main exposures, potential confounders, and maternal parenting outcomes are present in figure 2. Self-reported and filmed positive parenting outcomes were positively related with each other and with self-reported and filmed harsh parenting outcomes. PAFAS outcomes were all correlated among themselves, except for positive-encouragement and coercive practices. Filmed measures were also associated among themselves (positive interactions inversely related to coercive parenting). The highest correlations between the IPV variables and confounders were found with ACEs and maternal depressive symptoms. Maternal education was the variable that presented the highest correlations with positive parenting outcomes and filmed harsh parenting, whereas ACEs was the variable mostly associated with self-reported coercive behaviour.

Table 2 shows unadjusted and adjusted associations between recent IPV victimisation and maternal parenting outcomes when children were aged 4 years and 6–7 years. All unadjusted associations were significant between emotional IPV and physical or sexual IPV and lower maternal positive parenting scores for both direct observed and self-reported outcome measures. After adjusting for potential confounders (model 2), emotional IPV victimisation remained significantly associated with lower self-report positive encouragement (standardised mean difference [SMD] –0·09; 95% CI –0·18 to –0·01), poor parent–child relationship quality (SMD –0·15; 95% CI –0·24 to –0·06) and lower parental consistency (SMD –0·29; 95% CI –0·38 to –0·21) whereas physical or sexual IPV victimisation was associated with lower directly observed positive interactions (SMD –0·08; 95% CI –0·16 to 0·00), parent–child relationship quality (SMD –0·16; 95% CI –0·30 to –0·01) and parental consistency (SMD –0·27; 95% CI –0·41, –0·13). When depressive symptoms were included in the analysis (model 3), coefficients were attenuated but the associations between both subtypes of IPV and diminished parental consistency were retained, in addition to the association between emotional IPV and worse parent–child relationship. When children were aged 6–7 years, associations between IPV victimisation and self-reported parenting measures mirror those found at 4 years in the fully adjusted model.

**Table 2:** Unadjusted and adjusted associations between recent IPV victimisation when children are 4 years old and parenting outcomes at 4 years old and 6–7 years old. 2015 Pelotas Birth Cohort, Brazil

			**Emotional IPV**		**Physical or sexual IPV**	
			β* (95% CI)	p value	β* (95% CI)	p value
**Positive parenting**						
Children aged 4 years						
	Observed positive interactions*					
		Model 1 (unadjusted)	−0·08 (−0·13 to −0·02)	0·095	−0·17 (−0·25 to −0·09)	<0·0001
		Model 2	−0·05 (−0·10 to 0·01)	0·086	−0·08 (−0·16 to 0·00)	0·055
		Model 3 (model 2 + depressive symptoms)	−0·04 (−0·10 to 0·01)	0·15	−0·07 (−0·15 to 0·01)	0·10
	PAFAS positive encouragement					
		Model 1 (unadjusted)	−0·12 (−0·20 to −0·03)	0·0052	−0·18 (−0·31 to −0·05)	0·0064
		Model 2	−0·9 (−0·18 to −0·01)	0·035	−0·13 (−0·26 to 0·01)	0·071
		Model 3 (model 2 + depressive symptoms)	−0·08 (−0·16 to 0·01)	0·10	−0·10 (−0·24 to 0·03)	0·14
	PAFAS parent–child relationship					
		Model 1 (unadjusted)	−0·23 (−0·31 to −0·14)	<0·0001	−0·29 (−0·44 to −0·15)	<0·0001
		Model 2	−0·15 (−0·24 to −0·06)	0·0010	−0·16 (−0·30 to −0·01)	0·032
		Model 3 (model 2 + depressive symptoms)	−0·12 (−0·21 to −0·03)	0·011	−0·12 (−0·26 to 0·03)	0·11
	PAFAS parental consistency					
		Model 1 (unadjusted)	−0·35 (−0·43 to −0·26)	<0·0001	−0·41 (−0·54 to −0·28)	<0·0001
		Model 2	−0·29 (−0·38 to −0·21)	<0·0001	−0·27 (−0·41 to −0·13)	<0·0001
		Model 3 (model 2 + depressive symptoms)	−0·23 (−0·32 to −0·14)	<0·0001	−0·19 (−0·33 to −0·05)	0·0053
Children aged 6–7 years						
	PAFAS positive encouragement					
		Model 1 (unadjusted)	−0·09 (−0·17 to −0·01)	0·044	−0·13 (−0·27 to −0·01)	0·046
		Model 2	−0·05 (−0·14 to 0·03)	0·22	−0·08 (−0·22 to 0·05)	0·39
		Model 3 (model 2 + depressive symptoms)	−0·04 (−0·13 to 0·05)	0·39	−0·06 (−0·20 to 0·07)	0·65
	PAFAS parent–child relationship					
		Model 1 (unadjusted)	−0·17 (−0·26 to −0·08)	<0·0001	−0·27 (−0·41 to −0·12)	<0·0001
		Model 2	−0·13 (−0·21 to −0·04)	0·054	−0·15 (−0·30 to −0·00)	0·047
		Model 3 (model 2 + depressive symptoms)	−0·11 (−0·20 to −0·01)	0·023	−0·12 (−0·27 to 0·03)	0·12
	PAFAS parental consistency					
		Model 1 (unadjusted)	−0·34 (−0·43 to −0·25)	<0·0001	−0·45 (−0·59 to −0·31)	<0·0001
		Model 2	−0·29 (−0·38 to −0·19)	<0·0001	−0·32 (−0·47 to −0·18)	<0·0001
		Model 3 (model 2 + depressive symptoms)	−0·21 (−0·31 to −0·12)	<0·0001	−0·25 (−0·39 to −0·10)	0·0001
**Harsh parenting**						
Children aged 4 years						
	Observed coercive behaviour					
		Model 1 (unadjusted)	0·03 (−0·05 to 0·12)	0·43	0·04 (−0·09 to 0·17)	0·58
		Model 2	0·05 (−0·04 to 0·14)	0·26	0·06 (−0·08 to 0·20)	0·39
		Model 3 (model 2 + depressive symptoms)	0·05 (−0·04 to 0·14)	0·30	0·06 (−0·08 to 0·19)	0·42
	PAFAS coercive behaviour					
		Model 1 (unadjusted)	0·34 (0·26 to 0·42)	<0·0001	0·39 (0·27 to 0·52)	<0·0001
		Model 2	0·25 (0·17 to 0·34)	<0·0001	0·26 (0·13 to 0·38)	<0·0001
		Model 3 (model 2 + depressive symptoms)	0·18 (0·10 to 0·26)	<0·0001	0·18 (0·05 to 0·30)	0·0071
Children aged 6–7 years						
	PAFAS coercive behaviour					
		Model 1 (unadjusted)	0·35 (0·26 to 0·44)	<0·0001	0·45 (0·31 to 0·60)	<0·0001
		Model 2	0·28 (0·19 to 0·37)	<0·0001	0·34 (0·20 to 0·49)	<0·0001
		Model 3 (model 2 + depressive symptoms)	0·22 (0·13 to 0·31)	<0·0001	0·28 (0·13 to 0·42)	<0·0001

The outcomes are standardised (mean 0, SD 1). Model 1: unadjusted results. Model 2: adjusted for family income, maternal characteristics (age, education, ACEs, marital status, and parity), child characteristics (sex and age in months) and PIM/PIÁ participation. Model 3: adjusted for model 2 variables + maternal depressive symptoms (measured 2 years after delivery) and PIM/PIÁ participation. ACEs=adverse childhood experiences. IPV=intimate partner violence. PAFAS=Parenting and Family Adjustment Scales. PIÁ=The Pelotas Trial of Parenting Interventions for Aggression (which included ACT: Raising Safe Kids and a book-sharing programme). PIM=Primeira Infancia Melhor (Better First Childhood).

* Effect sizes (β) are standardised mean differences (95% CI). p values correspond to Wald test.

Associations between maternal IPV victimisation and harsh parenting outcomes showed that emotional and physical or sexual IPV were associated with increased self-reported coercive parenting but not with directly observed coercive parenting. Adjustment for confounders did not affect the associations significantly. Results from model 2 showed that IPV victimisation was associated with an average increase of 0·25 SD in coercive behaviours for both IPV indicators (emotional IPV [95% CI 0·17–0·33], p<0·001, physical or sexual IPV [95% CI 0·12–0·37], p=0·008) at age 4 years. At age 6–7 years, effect sizes were a little higher (0·28 [95% CI 0·19–0·37] for emotional IPV and 0·33 [95% CI 0·19–0·48] for physical or sexual IPV). When additionally accounting for depression, estimates were slightly attenuated but remained significant (p<0·001) at both timepoints.

After applying the Benjamini-Hochberg correction, all significant associations remained unchanged (appendix 2 p 15).

Results from the sensitivity analysis suggested that a dose–response relationship exists between the number of emotional IPV abusive acts and worse parent–child relationship quality at age 4 years and lower parental consistency at both ages 4 years and 6–7 years (appendix 2 p 16).

## Discussion

We investigated cross-sectional and longitudinal associations between maternal IPV victimisation and a broad range of parenting practices among mothers of young children in a Brazilian population-based cohort. We newly evaluated both self-reported maternal parenting practices, referring to general behaviours over an extended time frame (the previous month), and more nuanced aspects of mother–child interactions measured through direct observations. We were able to separate the potential distinct effects of emotional abuse and physical or sexual abuse. This distinction is important because, despite being the most common subtype of IPV, research on the consequences of emotional IPV has often been neglected or not carefully investigated, particularly independently of physical or sexual IPV.^29^ Robust associations were found between IPV victimisation (either emotional abuse or physical or sexual abuse) experienced by mothers when children were aged between 3 and 4 years and diminished parental consistency and increased coercive behaviour both when children were aged 4 years and 6–7 years, even after controlling for important confounders and accounting for earlier maternal depressive symptoms.

There were interesting differences in the findings from the direct observations compared with the maternal reports; this was particularly the case for coercive behaviour, for which associations greatly differed depending on the method of assessment. One potential explanation is that direct observations of mother–child interactions reflect only a snapshot, albeit more refined, of real-life family interactions, whereas self-reported parenting practices reflect more general maternal behaviour. There is evidence that maternal behaviours are affected by expectations in laboratory sessions in the presence of the researcher, and therefore mothers might exhibit less coercive behaviour. Another hypothesis is that harsh parenting manifests sporadically (eg, close in time to an experience of IPV), and this might be remembered by mothers, but not captured by the punctual observations. However, maternal reports could also be affected by mothers’ mental health status, leading them to report more difficulties in parenting. Although not necessarily measuring equivalent parenting behaviours, the use of validated and complementary measures can be considered a strength of the present study as no previous study has tested these associations with both types of measures.

There is growing international evidence on the intersections of IPV against women and violent discipline of children by caregivers within households.^8^ Consistent with our findings, associations between the use of harsh parenting and maternal violence victimisation have also been documented in previous studies, including some in LMICs.^4^, ^7^, ^8^, ^9^, ^10^ Although the question of why mothers experiencing IPV engage in more coercive parenting behaviour is beyond the scope of the current study, there is evidence suggesting that increased stress, anxiety, and emotional exhaustion could have an important role in mediating this process.^4^ There is also evidence indicating that women's harsh parenting practices can be influenced by their male partner's violent behaviour towards their children, as women can be motivated to keep children in line to avoid child abuse from partners.^11^ In light of this possibility, our analysis, which focuses exclusively on the dyadic relationship between mother and child, is limited by its omission of partner parental practices, thus leaving out an important aspect of the dynamic of parenting within violent contexts.

Our results showing negative associations between IPV victimisation and positive parenting outcomes extend the existing literature in LMICs that has primarily focused on negative parental practices such as harsh parenting.^8^ The set of positive parenting outcomes evaluated in our study includes various parenting practices, reflecting parenting that provides cognitive scaffolding, positive emotions of parents towards the child, engagement, and connectedness. In agreement with our findings, a recent mixed-method review showed that the sense of parenting self-efficacy and identity of mothers might be profoundly affected by the experience of IPV and compromise abilities to provide sufficient attachment, warmth, control, and monitoring.^4^ Qualitative studies show that IPV can undermine psychological capacities to interact with others and produce intense feelings of guilt and a sense of parental inadequacy, increasing challenges in providing nurturing care.^4^ The overall findings of the current study are also in line with the spillover theory, in which emotions and patterns of behaviours in marital conflicts are expected to influence parenting through increased harsh parenting and diminished warmth and affection.^6^

There are some limitations to be considered in this study. Under-reporting of experiences of violence is well described in the literature. Furthermore, IPV experiences beyond the previous year were not assessed in this cohort and this could contribute to dilute estimates as we are potentially including women who experienced IPV before the time frame assessed in our reference category. In this sense, further adjustment for maternal depressive symptoms is likely to also underestimate the magnitude of our estimates given the possibility that symptoms were a consequence of chronic IPV exposure. Another aspect to consider is that earlier IPV experiences (ie, before the 12-month reference period in the current study) might have resulted in relationship termination (and thus no more recent IPV), but could still have negatively influenced parenting outcomes and caused equal harm. We should be aware that, although not the focus of the current analysis, many pathways of resilience have been described in the literature.^6^ Future studies should investigate relevant moderators and mediators to help direct interventions. Finally, the findings of this study should be interpreted in the context of a single city cohort, which might not be generalisable to Brazil (eg, the sample might not be representative of the considerable socioeconomic variations present in the country).

The current study reveals that effects of IPV are specific to the domains of parenting captured in self-reports, rather than every aspect of parenting, even when considered at a more nuanced level. The findings add to the literature, pointing to the adverse consequences of IPV victimisation on mother–child dyadic relationships and underscoring the important role of interventions focused on improving caregiver's abilities to provide nurturing environments and parental practices (eg, reducing harsh parenting and increasing cognitive stimulation) that are more supportive for children exposed to violence within households. In this context, strategies addressing contextual obstacles to more positive parenting (eg, mental health) also seem warranted. Finally, IPV primary prevention interventions are crucial for improving maternal and child wellbeing.

## Data sharing

Applications to use the data can be made by contacting the researchers of the 2015 cohort (see http://www.epidemio-ufpel.org.br/site/content/faculty/ for a list of key faculty members) and completing the application form (http://www.epidemio-ufpel.org.br/site/content/studies/formularios.php). Researchers with successful applications will receive a dataset including the requested variables and unique participant identification.

## Declaration of interests

CVNC and JM report consulting fees as part of the *Lancet* Commission on gender-based violence and maltreatment of young people. All other authors declare no competing interests.

## Supplementary Materials

Portuguese translation of the abstract
