# Correction to *Lancet Glob Health* 2023; 11: e1393–401

**DOI:** 10.1016/S2214-109X(23)00427-8

**Published:** 2023-09-19

*Coll CVN, Barros AJD, Stein A, et al. Intimate partner violence victimisation and its association with maternal parenting (the 2015 Pelotas [Brazil] Birth Cohort): a prospective cohort study.* Lancet Glob Health *2023; **11:** e1393–401*—The funding information in the Summary was incomplete. This correction has been made as of Sept 19, 2023.

