# Supplementary material for: Intimate partner violence victimisation and its association with maternal parenting (the 2015 Pelotas [Brazil] Birth Cohort): a prospective cohort study
Source: Lancet Glob Health. 2023 Aug 15;11(9):e1393–401. doi: 10.1016/S2214-109X(23)00282-6 (PMC10447221; doi:10.1016/S2214-109X(23)00282-6)
Supplement: Portuguese translation of the abstract [file mmc1.pdf]

THE LANCET
Global Health

Supplementary appendix 1

This translation in Portuguese was submitted by the authors and we reproduce it as supplied. It has not been peer reviewed. *The Lancet*'s editorial processes have only been applied to the original in English, which should serve as reference for this manuscript.

Esta tradução em português foi submetida pelos autores e nós não fizemos quaisquer alterações. Esta versão não foi revista por pares. O processo editorial do The Lancet só foi aplicado à versão original em inglês, que deve servir como referência para este artigo.

Supplement to: Coll CVN, Barros AJD, Stein A, et al. Intimate partner violence victimisation and its association with maternal parenting (the 2015 Pelotas [Brazil] Birth Cohort): a prospective cohort study. *Lancet Glob Health* 2023; **11:** e1393–1401.

Histórico

A violência por parceiro íntimo (VPI) é altamente prevalente em países de baixa e média renda e tem sido um grande obstáculo para alcançar as metas globais de saúde para mulheres e crianças. Nosso objetivo foi investigar associações transversais e longitudinais entre a vitimização por VPI e as práticas parentais maternas de crianças pequenas em um estudo de coorte de nascimentos de base populacional no Brasil.

Métodos

A Coorte de Nascimentos de Pelotas de 2015 é uma coorte prospectiva em andamento, incluindo todos os nascimentos hospitalares ocorridos entre 1º de janeiro e 31 de dezembro de 2015, na cidade de Pelotas, Brasil. Quando as crianças tinham 4 anos de idade, as mães relataram vitimização por VPI emocional, física e sexual nos últimos 12 meses. Os desfechos parentais foram avaliados por meio de filmagens da mãe e da criança em tarefas interativas aos 4 anos de idade e entrevistas maternas aos 4 anos e 6-7 anos. Tarefas interativas foram filmadas nas instalações do Centro de Pesquisa Epidemiológica. Os resultados diretamente observados incluíram interações parentais negativas (por exemplo, coercitivas) e positivas (por exemplo, sensibilidade e reciprocidade), codificadas independentemente por uma equipe de psicólogos. A parentalidade autorreferida foi medida usando as subescalas de qualidade do relacionamento mãe-filho, encorajamento positivo, consistência parental e comportamento coercitivo do questionário *Parenting and Family Adjustment Scales*. Análises de regressão linear não ajustadas e ajustadas foram realizadas para avaliar as associações.

Achados

Dos 4,275 nascidos vivos inscritos na coorte, 3,730 díades mãe-filho foram incluídos em nossa amostra analítica aos 4 anos de idade e 3,292 aos 6-7 anos de idade. Após o ajuste para todos os possíveis fatores de confusão, a VPI emocional e a VPI física ou sexual foram associadas aos seguintes resultados parentais autorrelatados: pior qualidade do relacionamento mãe-filho (VPI emocional: p=0·011), menor consistência parental (VPI emocional: p <0·001, VPI física ou sexual: p=0·0053) e comportamento mais coercitivo (VPI emocional: p<0·001, VPI física ou sexual: p=0·0071) aos 4 anos de idade. Associações não foram observadas para encorajamento positivo autorrelatado e os desfechos parentais filmados no modelo com ajuste completo. Longitudinalmente, VPI aos 4 anos resultou em associações semelhantes quando as crianças tinham 6-7 anos, exceto entre VPI física ou sexual e qualidade do relacionamento mãe-filho.

Interpretação

Neste grande estudo de coorte, a vitimização materna por VPI foi consistentemente associada a uma pior relação mãe-filho, diminuição da consistência parental e aumento da parentalidade severa (coercitiva) relatada por mães de crianças pequenas. Assim como as iniciativas para prevenir a VPI, as intervenções parentais focadas em apoiar a capacidade dos cuidadores de fornecer um cuidado integral em estágios-chave no início do ciclo de vida são cruciais.
