# Supplementary material for: Intimate partner violence victimisation and its association with maternal parenting (the 2015 Pelotas [Brazil] Birth Cohort): a prospective cohort study
Source: Lancet Glob Health. 2023 Aug 15;11(9):e1393–401. doi: 10.1016/S2214-109X(23)00282-6 (PMC10447221; doi:10.1016/S2214-109X(23)00282-6)
Supplement: Supplementary appendix 2 [file mmc2.pdf]

THE LANCET
Global Health

Supplementary appendix 2

This appendix formed part of the original submission and has been peer reviewed. We post it as supplied by the authors.

Supplement to: Coll CVN, Barros AJD, Stein A, et al. Intimate partner violence victimisation and its association with maternal parenting (the 2015 Pelotas [Brazil] Birth Cohort): a prospective cohort study. *Lancet Glob Health* 2023; **11:** e1393–1401.
.

Annex Title	Page
Coding details of parental sensitivity and reciprocity in observed Book Sharing Task	1
Coding details of parental sensitivity and reciprocity in observed Don’t Touch Task	3
Table S1. Maternal parenting measures details and timing of assessment. 2015 Pelotas (Brazil) Birth Cohort Study	6
Table S2. Parenting and Family Adjustment Scales (PAFAS) items and coding in the 2015 Pelotas (Brazil) Birth Cohort Study	7
Table S3. Description of maternal parenting outcomes at 4 years and 6-7 years follow-ups.	10
Table S4. Description of maternal parenting outcomes according to intimate partner violence exposure status	11
Figure S1. Venn diagram depicting the overlap of types of intimate partner violence in the 2015 Pelotas (Brazil) Birth Cohort Study	12
Figure S2. DAG representation of assumptions of the relationships between variables under study.	13
Table S5. Unadjusted and adjusted associations between recent IPV victimization and observed positive interactions individual scores.	14
Table S6. Benjamini-Hochberg adjustment for multiple-testing.	15
Table S7. Unadjusted and adjusted associations between recent intimate partner violence victimisation (number of acts) and parenting outcomes. 2015 Pelotas (Brazil) Birth Cohort	16
References	18

Coding details of parental sensitivity and reciprocity in the observed *“Book Sharing Task”*

Sensitivity

Sensitivity refers to how responsive and well-attuned the parent is to the child’s signals and needs. Key categories in the coding scheme are as follows.

1. Highly Sensitive. This parent is highly attuned to child’s signals and responds to them promptly and appropriately. *She is able to see things from the child’s point of view; her perceptions of signals and communications are not distorted by her own agenda*. She ‘reads’ the child’s signals and communications skilfully, and knows what the meaning is of even subtle, minimal and understated cues. She nearly always gives the child what he/she indicates he/she wants, although not invariably so. When she feels that it is best not to comply with the demand – if, for example, the child is too excited, or wants something he/she should not have – she is tactful in acknowledging his communication and in offering an acceptable alternative. *She has ‘well-rounded’ interactions with the child, so that the transaction is smoothly completed and both she and the child appear satisfied. She makes her responses temporally contingent upon the child’s signals and communications.*

2. Sensitive. This parent also *interprets child’s communications accurately and responds to them promptly and appropriately* – but with less sensitivity than parents with a higher rating. She may be *less attuned to child’s more subtle behaviours* compared with the highly sensitive parent. She may be less skilful in dividing her attention between her child’s cues and competing demands, *and may sometimes miss subtle cues*. The child’s *clear and definite signals are, however, neither missed nor misinterpreted*. The parent empathises with the child and sees things from the child’s point of view. Her perceptions of the child’s behaviour are not distorted. Perhaps because her perception is less sensitive than that of other parents with a higher rating, her responses are not as consistently prompt or as finely appropriate. *Although there may be occasional little ‘mismatches’, the parent’s interventions are never seriously out of tune with child’s tempo, state and communications.*

3. Inconsistently Sensitive. Although this parent can be quite sensitive on occasion, there are some periods in which she is insensitive to her child's communication. She can be *sensitive at some times, or in respect to some aspects of the child's experience, but not in others*. Her awareness of the child and their interests and signals is intermittent. Her perception of the child's behaviour may also be distorted in regard to one or two aspects although it is accurate in others. She may be *prompt and appropriate in response to the child's communications at some times and in most respects, but either inappropriate or slow at times in other respects*. On the whole, however, she is *more frequently sensitive than insensitive, although the balance is not as positive as for a rating of 4*.

4. Insensitive. The parent frequently fails to respond to child's communications appropriately and/or promptly, although she may on some occasions show capacity for sensitivity in her responses to her child. Her insensitivity seems *linked to an inability to see things from the child's point of view*. She may be too frequently preoccupied with other things and therefore inaccessible to the child's signals and communications. She may also misperceive signals and interpret them inaccurately because of her own wishes. Furthermore, she may know well enough what the child is communicating but be disinclined to give him/her what he/she wants. *She may delay an otherwise appropriate response to such an extent that it is no longer contingent on her child's signal, and indeed perhaps is no longer appropriate to his state, mood or activity*. This parent may also respond with seeming appropriateness to child's communications but break off the transactions before the child is satisfied, so that their interactions seem fragmented and incomplete or her responses are perfunctory, or impatient. Despite such clear evidence of insensitivity, however, this parent is not as consistently insensitive as the parent with a rating of 1. This parent *can modify her own behaviour and goals and can show some sensitivity in her handling of the child, either when the child's wishes, moods and activity are not too deviant from the parent's wishes and moods, or when the child is truly distressed or otherwise communicating very forcefully and compellingly*.

5. Highly Insensitive. The extremely insensitive parent seems geared almost exclusively to her own wishes, moods and activity, i.e. parent's interventions and initiations of interaction are *determined largely by her own interests and agenda*; if they mesh with the child's signals, this is often no more than coincidence. This is not to say that the parent *never* responds to the child's signals, as occasionally she does if these signals are intense enough, prolonged enough or repeated often enough. However, responses are generally either delayed or inappropriate (e.g., rejecting the child's attempt to go backwards in the book). Since there is usually a disparity between the insensitive parent's own wishes and activity and the child's signals, the parent typically ignores, rejects or else distorts the meaning of the child's behaviour. Thus, when the parent *does* respond to her child's signals, *her response is characteristically inappropriate in kind, or fragmented and incomplete*. Such insensitive behaviour occurs throughout the interaction.

Reciprocity

This refers to the extent of **mutual interchange** between parent and child. The child's input is received and responded to by the parent and vice versa. In particular, there is joint orientation and co-ordination of the actions between the parent and the child in achieving a goal. The shared co-ordination and turn taking makes a very important contribution to this dimension. Referencing would be expected to be in higher reciprocal interaction, although the dyad may be so absorbed in sharing actions that there is no need for sharing the gaze as well.

1. Very Much Reciprocity. The entire session is characterised by reciprocal interaction, exchanges are mutual, finely tuned, co-ordinated and smooth.

2. Much Reciprocity. Much of the session is characterised by reciprocal interaction, most of the exchanges are mutual and characterised by turn taking, and there is joint engagement; there may be some occasions when the parent and child are not engaged together.

3. Moderate Reciprocity. The session is half the time characterised by reciprocal interaction.

4. Little Reciprocity. Shared interaction occurs very little, and only occasionally do the parent and child follow each other's suggestions. Turn taking is rare. It is rare for the parent and child to be focused on the same aspects of the experience of the book, or for them to co-ordinate their engagement around the book.
5. No Reciprocity. Hardly any reciprocity is observed; there is no turn taking or shared communication, the parent and child engage in different things at a given time and co-ordination is never or hardly ever observed.

Coding details of parental guidance and coercive behaviour in the observed *"Don't Touch Task"*

Guidance, Verbal (Scale). This measure concern techniques such as reasoning, providing distractions (e.g., re-directing child's attention to another object or behaviour, e.g., naming or counting); positive incentives (e.g., telling child they can play later); suggestions; explanation for delay for "don't touch"; induction (explanation about norms), and mild prompts. Note, reasons and explanations are considered high quality support. If distraction and reasons are given in the context of a threat, do **not** count as guidance, but count as a verbal threat- e.g., 'Do you see that lady with the camera-she will be angry with you'. If a verbal threat is used, and is then followed by a positive verbal guidance, or *vice versa*, within the same time block, code both. If the utterance is **ambiguous**- e.g., 'Don't cry, *we will leave'*, do not give credit, but also do not score as a verbal threat. Note, tone of voice needs to be taken into account, with the code downgraded for harsh tone, and upgraded if more positive – e.g., singing.

0 = carer shows no use of verbal support-guidance

1 = carer uses one or at most two mild prompts- never harsh, or *very* briefly uses a verbal distraction, or mention of a later positive activity, or briefly engages in a verbal activity, or makes a single, isolated, brief mention of getting the toy later 'e.g., 'you'll get it'

Examples: 'Wait, don't rush, look, look'.

'Wait don't touch, please don't touch'

'Come, don't touch the toys'

'You'll play later'

2 = carer distracts child or engages them in a verbal activity more than just briefly, but not all the time- they might draw the child's attention to something in the room or engage them in a song or counting; they may briefly mention the task element, they may use mild prompts, they may make more than one brief mention of getting the toy later. Verbal support may be accompanied by a physical action, like a point, or like moving child during a song, but if the verbal element predominates code only the verbal code. Note, if the physical element is just as predominant- e.g., having a conversation about the child's shoe while moving it around and physically exploring it, a physical support code can also be given.

Examples: 'Look at your foot, show me your foot, and another one'

'Wait, wait/ don't touch it/ Yo, Yo, let's count, first, say 1'

'Wait, you will get it soon/ you will get it soon/ leave it where it is.

'Will you play with them/do you know how to play with them?'

'Look there, she is filming with the camera/she is very busy filming'

3 = carer reasons and explains the task to the child, or provides constant engaging distraction throughout. If any negative component is included, downgrade to a 2.

Examples: 'Wait, the lady will give it to you soon, OK. Look first what the lady is doing, she is taking a film, look there. Isn't the mother beautiful? She's beautiful, isn't she?'

'The lady will bring other things first/She is coming soon, soon/say, now, look at the lady, she's taking films'.

(child: give me the toys, mum) 'These ones and this one, leave them like that, the lady's tempting us/she's tempting us/leave her with her toys.'

'Leave the lady's toys, let's ignore her./Let's ignore her with her toys/this one is for the lady, and this one./Which one do you want to play with?.'

'Don't touch them (affectionate reminder)/Do you want to play? Wait/ mom will give them to you.'

Guidance, Physical (Scale): includes playing physical games or engaging in other non-forceful activity, mainly to occupy and distract the child and support them through the task. If carer uses verbal support/guidance, and it has a clear physical focus, then it can also be coded in the physical support ratings.

0 = no attempt to engage child physically, or only a very brief physical movement

1 = brief attempt at a physical game, or low-level repetitive motion for more than half time- e.g., gently jiggling/rocking child on lap, or affectionate physical contact that supports the task, or that attempts to comfort the child, or brief physical- but not verbal- pointing to the room. Note a brief affectionate contact, like giving a kiss, if it seems to be done as a distraction is rated here. If carer engages with child physically through more than half the task, but without any indication that the activity is geared to distracting the child, count as 1 rather than 2 (e.g., ID who rubs child's trouser to remove a mark)

2 = carer distracts or engages child in a physical game or activity more than just briefly- e.g., engaging them in clapping/dancing but not for more than half the time, or repeated use of gesture-

but not words- to engage/distract the child, or gives active engaged comfort if the child is distressed.

3 = carer distracts or engages child in a physical game or activity- e.g., clapping/dancing, for most/all the time, pointing to things to entertain most of time/throughout.

Coercive, harsh, control/discipline Physical: This behaviour involves power assertion and the use of force. It includes physically enforcing child behaviour; forcibly taking toys away from the the child; restraining or holding the child forcibly; spanking; slapping; grabbing; holding child's face forcibly; pulling child's arm (hard), imposing an action on the child; turning child forcibly, making threatening gesture; showing anger.

0 = carer shows no use of force or physical restraint throughout the time block. Can include brief, light, physical contact to remind child- e.g., placing hand lightly on arm. Can include straightening child, placing them in a more comfortable position. Can include a physical game that inevitably involves physical contact, but which is done without any hint of restraint.

1= carer shows either one brief episode of clear, but not strong, physical restraint (e.g., pulling child away from toys, or stopping them moving towards them, but not with clear force), or prolonged mild restraint (e.g., holding arms around child so that they cannot move towards toys, but not holding tight; or resists child's attempts to push parent's arm/hand away), or plays a game that is clearly somewhat restraining.

2= carer shows either one brief episode of clear, strong, physical force (e.g., pulling child away forcibly), or several instances of clear, but not strong physical restraint, or else prolonged moderate force without aggression (e.g., holding arms tightly around child so that they cannot move towards toys).

3 = carer shows one episode of clear directed aggression (e.g., smack, hit, clear threat gesture, holding child's face forcibly), or more than one episode, or prolonged use, of clear strong physical restraint, not necessarily accompanied by clear directed aggression (e.g., forceful pulling, gripping with strong force, or very tightly holding arms around the child).

Coercive, harsh, control/discipline Verbal (Event count): this behaviour involves power assertion and use of verbal, but not physical force. It includes criticisms, threats, angry commands, shouting, harsh, irritated voice.

Table S1. Maternal parenting measures details and timing of assessment. 2015 Pelotas (Brazil) Birth Cohort Study

Outcome	Measure	Details	Score range*. "Desirable" score is higher (+) or lower (-)	4 Years Follow-Up	6-7 Years Follow-Up
**Positive parenting**	**Observed positive interactions**<br>Combined score of:<br>• Filmed Responsive Interactions – sensitivity<br>• Filmed book-sharing - sensitivity<br>• Filmed book-sharing task – reciprocity<br>• Filmed Don't touch – Guidance	See individual measures below for details. The final combined score was calculated as the mean of the z-score on each task.	z-score (+)	✓	
	**Filmed Book-sharing task – sensitivity***<br>Filmed Book-sharing task – parental sensitivity score (Cooper et al., 2014; [1] Murray et al., 2016 [2])	Mother and child are filmed for approximately 5 minutes without examiner interference while looking at a picture book together. Subsequently, reviewers study the filmed interaction and code mother's behaviour in terms of awareness of the child's focus of interest and sensitive responses. Variables are derived on a 5-point Likert scale: 1 (highly insensitive) – 5 (highly sensitive). More details on the coding can be found in the Appendix.<br>Weighted Kappa: 0.98	1-5 (+)	✓	
	**Filmed Book-sharing task – reciprocity***<br>Filmed Book-sharing task – parent-child reciprocity score (Cooper et al., 2014; [1] Murray et al., 2016 [2])	Mother and child are filmed for approximately 5 minutes without examiner interference while looking at a picture book together. Subsequently, reviewers study the filmed interaction and code mother's behaviours in terms of engagement in reciprocal exchanges with the child. Variables are derived on a 5-point Likert scale: 1 (no reciprocity) – 5 (high reciprocity). More details on the coding can be found in the Appendix.<br>Weighted Kappa: 0.98	1-5 (+)	✓	

Outcome	Measure	Details	Score range*. "Desirable" score is higher (+) or lower (-)	4 Years Follow-Up	6-7 Years Follow-Up
	**Filmed Responsive Interactions**<br>Responsive Interactions task – overall responsive interactions score (Schneider et al., 2021 [3])	A rapid assessment of the extent to which a parent identifies and responds, incorporating sensitivity and stimulation, to the feelings and thoughts of the child. Measures, three interconnected skills of the caregiver (i) communicative clarity (providing meaningful verbal/nonverbal inputs to the child and fostering of shared understanding of the goals of the task); (ii) mindreading (taken into account what the child knows and understands) and (iii) mutuality building (promoting reciprocity through a challenging task that elicits cooperation). The mother is instructed to sit and play with her child for five minutes constructing a robot as per pictures shown, using play (Lego/Duplo type blocks). Trained psychologits code 11 items on a 5-point Likert scale, and the mean represents the total responsive interactions score. Further details on the items composing the instrument and how behaviors are coded can be found in elsewhere (Schneider et al., 2021)<br>Intra-class correlation coefficient (ICC): 0.62	1-5 (+)	✓	
	**Filmed Don't Touch task – guidance****<br>Parental guidance on filmed 'Don't touch' task (Kochanska &Aksan, 1995 [4])	During the Don't Touch task toys are placed in front of the child and the child is prohibited from touching the toys for three minutes. After the task is finished, the examiner allows the child to play with the toys for a few minutes, until the next task is presented. Maternal supportive physical and verbal guidance is coded for each 20 second period during the tasks, and the average represents the final score on each task. See the Appendix for more details on the coding.<br>Weighted Kappa: 0.99	0-3 (+)	✓	
	**PAFAS - positive encouragement**<br>PAFAS questionnaire positive encouragement subscale (Sanders et al, 2014; [5] Santana et al, 2018 [6])	Questionnaire completed by the mother. Two items of the positive encouragement subscale are summed to form a final score ("I praise my child when they behave well" and "I give my child attention (e.g. hug, wink, smile or kiss) when they behave well"). Each item is rated on a 4-point scale from 0 (not true of me at all) to 3 (true of me very much).	0-6 (+)	✓	✓

Outcome	Measure	Details	Score range*. "Desirable" score is higher (+) or lower (-)	4 Years Follow-Up	6-7 Years Follow-Up
	**PAFAS – parent-child relationship quality** PAFAS questionnaire parent-child subscale (Sanders et al, 2014; [5] Santana et al, 2018 [6])	Questionnaire completed by the mother. Five items of the relationship sub-scale are summed to form a final score ("I chat/ talk with my child", "I enjoy giving my child hugs, kisses and cuddles", "I am proud of my child", "I enjoy spending time with my child" and "I have a good relationship with my child". Each item is rated on a 4-point scale from 0 (not true of me at all) to 3 (true of me very much).	0-15 (+)	✓	✓
	**PAFAS – consistency** PAFAS questionnaire parental consistency subscale (Sanders et al, 2014; [5] Santana et al, 2018 [6])	Questionnaire completed by the mother. Three items of the relationship sub-scale are summed to form a final score ("If my child doesn't do what they're told to do, I give in and do it myself", "I threaten something (e.g. turn of TV) when my child misbehaves but I don't follow through" and "I give my child what they want when they get angry or upset". Each item is rated on a 4-point scale from 0 (not true of me at all) to 3 (true of me very much).	0-9 (+)	✓	✓
**Harsh parenting**	**PAFAS – coercive behaviour** PAFAS questionnaire parental coercion subscale (Sanders et al, 2014; [5] Santana et al, 2018 [6])	Questionnaire completed by the mother. Four items on the coercion subscale are summed to form a final score ("I shout or get angry with my child when they misbehave", "I try to make my child feel bad (e.g. guilt or shame) for misbehaving to teach them a lesson", "I spank (smack) my child when they misbehave" and "I get annoyed with my child".	0-12 (-)	✓	✓
	**Filmed Don't Touch task – observed coercive behaviour**** Combined score of: • Parental verbal and physical coercion on filmed 'Don't touch' task (Kochanska & Aksan, 1995 [4])	During the Don't Touch task toys are placed in front of the child and the child is prohibited from touching the toys for three minutes. Maternal coercive behaviour (verbal threat and/or the degree of physical coercive control is coded for each 20 second period during the tasks. An overall score is derived by the count of coercive acts by for the entire period of the activity. See the Appendix for more details on the coding.	0-3 (-)	✓	

*The book used was the "A Day in the Park" — a book without text, developed by the research staff to represent local activities and include content that may elicit dialogue of particular interest for the research project (e.g., children playing and conflict breaking out over toys in a sandpit). **The data on maternal guidance in the Don't Touch task were analysed without reference to children's own behaviour, which may evoke differential maternal behaviours.

Table S2. Parenting and Family Adjustment Scales (PAFAS) items and coding in the 2015 Pelotas (Brazil) Birth Cohort Study.

Subscales	Items	Score range	Interpretation*
**Parental consistency**	1. If my child doesn't do what they're told to do, I give in and do it myself	**0-9**	Higher scores indicate higher level of consistency
	2. I threaten something (e.g. turn of TV) when my child misbehaves but I don't follow through		
	3. I give my child what they want when they get angry or upset		
**Coercive parenting**	4. I shout or get angry with my child when they misbehave	**0-12**	Higher scores indicate higher level of coercive parenting
	5. I try to make my child feel bad (e.g. guilt or shame) for misbehaving to teach them a lesson		
	6. I spank (smack) my child when they misbehave		
	7. I get annoyed with my child		
**Positive encouragement**	8. I praise my child when they behave well	**0-6**	Higher scores indicate higher level of positive encouragement
	9. I give my child attention (e.g. hug, wink, smile or kiss) when they behave well		
**Parent-child relationship**	10. I chat/ talk with my child	**0-15**	Higher scores indicate higher level of parent-child relationship
	11. I enjoy giving my child hugs, kisses and cuddles		
	12. I am proud of my child		
	13. I enjoy spending time with my child		
	14. I have a good relationship with my child		

*For the purposes of our analysis, parental consistency, positive encouragement, and parent-child relationship scores were reversed so that a higher score indicates higher levels of positive parenting. A validation study was carried out on the 18-item parenting subscale using data from the 30-item short version of PAFAS, applied to the 4-year follow-up of the 2015 Cohort (N=3883). The results of exploratory and confirmatory factor analysis indicated that four items of the scale do not show good fit. Two items from consistency subscale, one item from coercive subscale and one from positive encouragement subscale were excluded. Therefore, the final validated scale has 14 items, and showed a good reliability coefficient (0.912). The results are in accordance with the validation study of the PAFAS scale in its full version (40 items), carried out by Santana (2018) with 315 mothers of pre-school children, aged between three and five years from Mato Grosso do Sul/Brazil. In Santana's study, the same 14-items that showed good fit in our model remained valid.

Table S3. Description of maternal parenting outcomes at 4 years and 6-7 years follow-ups**.**

**Parenting variables (original scale)**	Mean (SD)	Median (IQR)	Range (min-max)
**Children aged 4y**			
**Positive parenting**			
Observed Positive interactions	2.60 (0.59)	2.63 (2.21; 3.02)	0-4.55
PAFAS Positive encouragement	5.20 (0.97)	6 (4; 6)	0-6
PAFAS Parent-child relationship	8,72 (1.83)	10 (8; 10)	0-10
PAFAS Parental consistency	6.97 (1.74)	7 (6; 8)	0-9
**Harsh parenting**			
Observed Coercive behaviour	0.59 (0.76)	1 (0; 1)	0-3
PAFAS Coercive behaviour	3.65 (2.01)	3 (2; 5)	0-12
**Positive parenting (intermediate variables)***			
Observed Parent sensitivity	3.63 (0.85)	4 (3; 4)	1-5
Observed Responsive interactions	2.54 (0.82)	2.55 (1.91; 3.09)	1-4.72
Observed Guidance	0.68 (0.43)	0,69 (0.31; 1)	0-2.44
Observed Reciprocity	3.56 (1.08)	4 (3; 4)	0-5
**Children aged 6-7y**			
**Positive parenting**			
PAFAS Positive encouragement	5.40 (0.89)	6 (5; 6)	0-6
PAFAS Parent-child relationship	8.97 (1.71)	10 (9; 10)	0-10
PAFAS Parental consistency	6.73 (1.80)	7 (6; 8)	0-9
**Harsh parenting**			
PAFAS Coercive Behaviour	3.19 (1.96)	3 (2; 4)	0-12

Filmed activities N varies between 3588 and 3606
Questionnaires N at children aged 4y varies between 3707 and 3715. Questionnaires N of children aged 6-7y is 3292
* Intermediate variables used to create the observed positive interactions score

Table S4. Description of maternal parenting outcomes according to intimate partner violence exposure status.

Parenting variables (standardized)	Intimate partner violence<br>No<br>(n=2726)	 Emotional<br>(n=766)	 Physical and/or sexual<br>(n=283)
	Mean (SD)		
**Children aged 4y**			
**Positive parenting**			
Observed Positive interactions	0.04 (0.70)	-0.04 (0.70)	-0.13 (0.68)
PAFAS Positive encouragement	0.03 (0.99)	-0.09 (1.02)	-0.15 (1.05)
PAFAS Parent-child relationship	0.06 (0.95)	-0.17 (1.11)	-0.24 (1.19)
PAFAS Parental consistency	0.08 (0.96)	-0.27 (1.07)	-0.32 (1.08)
**Harsh parenting**			
Observed Coercive behaviour	-0.01 (0.99)	0.03 (1.03)	0.03 (1.06)
PAFAS Coercive behaviour	-0.05 (0.97)	0.29 (1.01)	0.34 (1.01)
**Positive parenting (intermediate variables)***			
Observed Parent sensitivity	0.04 (1.00)	-0.04 (0.97)	-0.14 (0.95)
Observed Responsive interactions	0.05 (0.98)	-0.06 (1.01)	-0.15 (0.95)
Observed Guidance	0.02 (0.99)	-0.02 (1.01)	-0.16 (1.04)
Observed Reciprocity	0.04 (0.99)	-0.02 (1.01)	-0.09 (1.01)
**Children aged 6-7 years**			
**Positive parenting**			
PAFAS Positive encouragement	0.06 (0.95)	-0.03 (1.02)	-0.07 (1.01)
PAFAS Parent-child relationship	0.09 (0.92)	-0.08 (1.03)	-0.18 (1.11)
PAFAS Parental consistency	0.06 (0.96)	-0.28 (1.07)	-0.39 (1.06)
**Harsh parenting**			
PAFAS Coercive behaviour	-0.03 (0.96)	0.31 (1.04)	0.42 (1.11)

Observed (filmed) activities N varies between 3588 and 3606
Questionnaires N at children aged 4y varies between 3707 and 3715. Questionnaires N at children aged 6-7y is 3292.
* Intermediate variables used to create the Observed Positive interactions outcome.

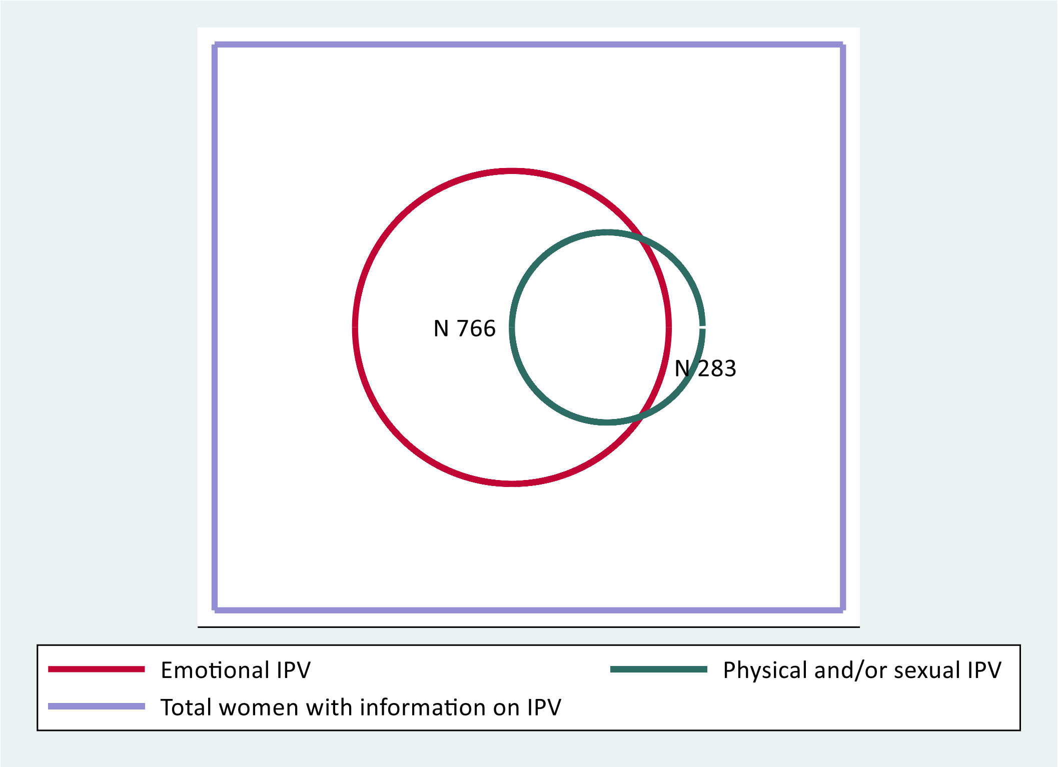

Figure S1. Venn diagram depicting the overlap of types of intimate partner violence in the 2015 Pelotas Birth Cohort Study.

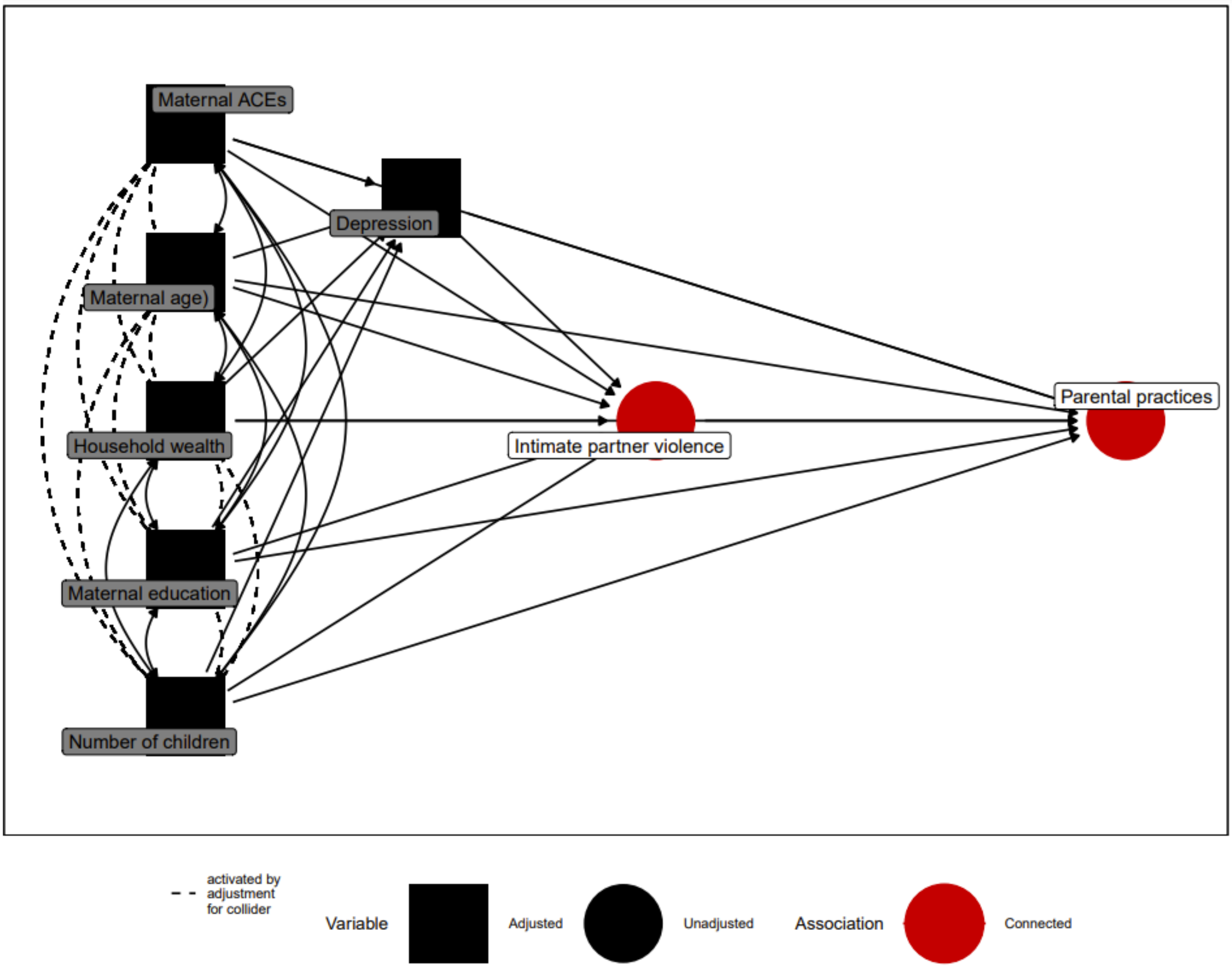

Figure S2. DAG representation of assumptions of the relationships between variables under study.

Table S5. Unadjusted and adjusted associations between recent IPV victimization and observed positive interactions individual scores.

**Maternal parenting outcomes**	**Emotional IPV**		**Physical and/or sexual IPV**	
	β (95% CI)	p-value	β (95% CI)	p-value
**Guidance**				
Model 1 (unadjusted)	-0.04 (-0.12; 0.04)	0.32	-0.18 (-0.31; -0.05)	0.01
Model 2	-0.01 (-0.09; 0.08)	0.90	-0.07 (-0.20; 0.06)	0.27
Model 3 (Model 2 + depressive symptoms)	0.01 (-0.08; 0.09)	0.90	-0.06 (-0.18; 0.07)	0.39
**Responsive interactions**				
Model 1 (unadjusted)	-0.11 (-0.19; -0.03)	0.01	-0.20 (-0.32; -0.08)	0.001
Model 2	-0.07 (-0.15; 0.01)	0.09	-0.09 (-0.21; 0.02)	0.12
Model 3 (Model 2 + depressive symptoms)	-0.06 (-0.14; 0.02)	0.15	-0.07 (-0.19; 0.04)	0.22
**Reciprocity**				
Model 1 (unadjusted)	-0.06 (-0.14; 0.02)	0.16	-0.12 (-0.25; 0.00)	0.05
Model 2	-0.05 (-0.14; 0.03)	0.23	-0.06 (-0.19; 0.07)	0.34
Model 3 (Model 2 + depressive symptoms)	-0.05 (-0.14; 0.03)	0.23	-0.06 (-0.19; 0.07)	0.34
**Sensitivity**				
Model 1 (unadjusted)	-0.08 (-0.16; -0.00)	0.04	-0.18 (-0.30; -0.06)	0.002
Model 2	-0.06 (-0.14; 0.02)	0.16	-0.09 (-0.22; 0.03)	0.14
Model 3 (Model 2 + depressive symptoms)	-0.05 (-0.13; 0.03)	0.23	-0.08 (-0.21; 0.04)	0.20

These four intermediate scores were used to create the Observed positive interactions outcome.

β are standardised mean differences. p-values correspond to Wald test.

Model 2: adjusted for family income at age 2-y, maternal characteristics (age, education, ACEs, parity and having a partner), child characteristics (sex and age in months) and PIM/PIÁ participation.

Model 3: adjusted for Model 2 variables + maternal depressive symptoms at age 2-y.

Table S6. Benjamini-Hochberg adjustment for multiple-testing.

Parenting outcomes at 4y follow-up			Parenting outcomes at 6y follow-up	
Rank	Original *P* value	*Q* value	Original *P* value	*Q* value
1	**<0.001**	**0.004166667**	**<0.001**	**0.00625**
2	**<0.001**	**0.008333333**	**<0.001**	**0.0125**
3	**<0.001**	**0.0125**	**<0.001**	**0.01875**
4	**<0.001**	**0.016666667**	**<0.001**	**0.025**
5	**<0.001**	**0.020833333**	**<0.001**	**0.03125**
6	**<0.001**	**0.025**	**<0.001**	**0.0375**
7	**0.001**	**0.029166667**	**<0.001**	**0.04375**
8	**0.007**	**0.033333333**	**<0.001**	**0.05**
9	**0.008**	**0.0375**	**0.001**	**0.05625**
10	**0.011**	**0.041666667**	**0.005**	**0.0625**
11	**0.032**	**0.045833333**	**0.044**	**0.06875**
12	**0.035**	**0.05**	**0.046**	**0.075**
13	0.055	0.054166667	0.124	0.08125
14	0.065	0.058333333	0.219	0.0875
15	0.084	0.0625	0.393	0.09375
16	0.095	0.066666667	0.645	0.1
17	0.102	0.070833333		
18	0.115	0.075		
19	0.142	0.079166667		
20	0.150	0.083333333		
21	0.262	0.0875		
22	0.296	0.091666667		
23	0.390	0.095833333		
24	0.424	0.1		

P values of adjusted models (2 and 3) were considered for Benjamini-Hochberg adjustment. A false discovery rate of 10% (0.1) was used.
Q-value is the Benjamini-Hochberg critical value.[7]
The largest *P* value that has *P* < Benjamini-Hochberg critical value is significant, and *all* of the *P* values smaller than it are also significant. The results presented in the paper did not change after Benjamini- Horchberg correction (in bold)

Table S7. Unadjusted and adjusted associations between recent intimate partner violence victimisation (number of acts) and parenting outcomes. 2015 Pelotas (Brazil) Birth Cohort

	**Emotional IPV**		
	Model 1 (unadjusted)	Model 2	Model 3 (Model 2 + depressive symptoms)
	β (95% CI)	β (95% CI)	β (95% CI)
**Positive parenting**			
**Children aged 4y**			
**Observed Positive interactions* 4y**	p=0.023	p=0.16	p=0.26
0	Ref.	Ref.	Ref.
1	-0.05 (-0.13; 0.02)	-0.03 (-0.10; 0.04)	-0.02 (-0.09; 0.05)
2+	-0.10 (-0.18; -0.02)	-0.07 (-0.14; 0.01)	-0.06 (-0.14; 0.01)
**PAFAS Positive encouragement 4y**	p=0.02	p=0.10	p=0.22
0	Ref.	Ref.	Ref.
1	-0.10 (-0.20; 0.01)	-0.10 (-0.21; 0.00)	-0.09 (-0.20; 0.02)
2+	-0.14 (-0.26; -0.02)	-0.08 (-0.20; 0.04)	-0.05 (-0.18; 0.07)
**PAFAS Parent-Child relationship 4y**	p<0.001	p=0.003	p=0.032
0	Ref.	Ref.	Ref.
1	-0.16 (-0.26; -0.05)	-0.11 (-0.22; -0.01)	-0.09 (-0.20; 0.02)
2+	-0.31 (-0.44; -0.18)	-0.20 (-0.33; -0.07)	-0.15 (-0.29; -0.02)
**PAFAS Parental consistency 4y**	p<0.001	p<0.001	p<0.001
0	Ref.	Ref.	Ref.
1	-0.30 (-0.41; -0.19)	-0.26 (-0.37; -0.16)	-0.21 (-0.32; -0.10)
2+	-0.41 (-0.53; -0.29)	-0.33 (-0.46; -0.21)	-0.25 (-0.38; -0.12)
**Children aged 6-7y**			
**PAFAS Positive encouragement 6-7y**	p=0.13	p=0.48	p=0.69
0	Ref.	Ref.	Ref.
1	-0.09 (-0.20; 0.02)	-0.06 (-0.17; 0.05)	-0.05 (-0.16; 0.06)
2+	-0.08 (-0.20; 0.04)	-0.05 (-0.18; 0.08)	-0.03 (-0.16; 0.10)
**PAFAS Parent-Child relationship 6-7y**	p<0.001	p=0.02	p=0.062
0	Ref.	Ref.	Ref.
1	-0.18 (-0.29; -0.07)	-0.14 (-0.25; -0.03)	-0.13 (-0.24; -0.01)
2+	-0.16 (-0.28; -0.03)	-0.10 (-0.23; 0.03)	-0.08 (-0.21; 0.05)
**PAFAS Parental consistency 6-7y**	p<0.001	p<0.001	p<0.001
0	Ref.	Ref.	Ref.
1	-0.26 (-0.38; -0.15)	-0.21 (-0.33; -0.10)	-0.16 (-0.27; -0.04)
2+	-0.44 (-0.56; -0.31)	-0.38 (-0.51; -0.25)	-0.29 (-0.42; -0.16)
**Harsh parenting**			
**Children aged 4y**			
**Observed Coercive behaviour 4y**	p=0.56	p=0.30	p=0.33
0	Ref.	Ref.	Ref.
1	0.01 (-0.10; 0.11)	0.01 (-0.10; 0.12)	0.01 (-0.10; 0.12)
2+	0.06 (-0.05; 0.18)	0.10 (-0.03; 0.23)	0.10 (-0.03; 0.22)
**PAFAS Coercive behaviour 4y**	p<0.001	p<0.001	p<0.001
0	Ref.	Ref.	Ref.
1	0.30 (0.19; 0.40)	0.22 (0.11; 0.32)	0.16 (0.05; 0.26)

2+	0.39 (0.27; 0.50)	0.30 (0.18; 0.42)	0.21 (0.09; 0.33)
**Children aged 6-7y**			
**PAFAS Coercive behaviour 6-7y**	p<0.001	p<0.001	p<0.001
0	Ref.	Ref.	Ref.
1	0.32 (0.21; 0.43)	0.24 (0.13; 0.36)	0.20 (0.08; 0.31)
2+	0.38 (0.26; 0.51)	0.33 (0.20; 0.45)	0.25 (0.13; 0.38)

Results are mean differences (95% CI). p-values correspond to Wald test.
IPV is categorized in 0, 1, 2 or more episodes.
The outcomes are standardized (mean 0, SD 1).
Model 1: unadjusted results
Model 2: adjusted for family income, maternal characteristics (age, education, ACEs and parity) and PIM/PIÁ participation
Model 3: adjusted for Model 2 variables + maternal depressive symptoms (measured 2 years after delivery) and PIM/PIÁ participation

References

1. Cooper PJ, Vally Z, Cooper H, Radford T, Sharples A, Tomlinson M, et al. Promoting Mother-Infant Book Sharing and Infant Attention and Language Development in an Impoverished South African Population: A Pilot Study. Early Child Educ J. 2014;42(2). doi: 10.1007/s10643-013-0591-8.

2. Murray L, De Pascalis L, Tomlinson M, Vally Z, Dadomo H, MacLachlan B, et al. Randomized controlled trial of a book-sharing intervention in a deprived South African community: effects on carer–infant interactions, and their relation to infant cognitive and socioemotional outcome. J Child Psychol Psychiatry Allied Discip. 2016;57(12). doi: 10.1111/jcpp.12605.

3. Schneider A, Rodrigues M, Falenchuk O, Munhoz TN, Barros AJD, Murray J, et al. Cross-cultural adaptation and validation of the brazilian portuguese version of an observational measure for parent–child responsive caregiving. Int J Environ Res Public Health. 2021;18(3). doi: 10.3390/ijerph18031246.

4. Kochanska G, Aksan N. Mother-Child Mutually Positive Affect, the Quality of Child Compliance to Requests and Prohibitions, and Maternal Control as Correlates of Early Internalization. Child Dev. 1995;66(1). doi: 10.1111/j.1467-8624.1995.tb00868.x.

5. Sanders MR, Morawska A, Haslam DM, Filus A, Fletcher R. Parenting and family adjustment scales (PAFAS): Validation of a brief parent-report measure for use in assessment of parenting skills and family relationships. Child Psychiatry Hum Dev. 2014;45(3). doi: 10.1007/s10578-013-0397-3.

6. Santana LR. Adaptação Transcultural e Validação da Parenting and Family Adjustment Scales. [Internet]. Universidade Federal de Dourados; 2018.

7. Benjamini Y, Hochberg Y. Controlling the False Discovery Rate: A Practical and Powerful Approach to Multiple Testing. J R Stat Soc Ser B. 1995;57(1). doi: 10.1111/j.2517-6161.1995.tb02031.x.
